# Effectiveness of Ayurveda Intervention in the Management of Infertility: A Systematic Review

**DOI:** 10.7759/cureus.57730

**Published:** 2024-04-06

**Authors:** Isha Rathi, Aarushi Mavi, Mohd Shannawaz, Shazina Saeed, Ankur Yadav, Shamimul Hasan

**Affiliations:** 1 Epidemiology and Public Health, Amity Institute of Public Health & Hospital Administration, Amity University, Noida, IND; 2 Communication, National Institute of Health and Family Welfare, New Delhi, IND; 3 Oral Medicine and Radiology, Jamia Millia Islamia, New Delhi, IND

**Keywords:** public health, systematic literature review, ayurveda curative methods, ayurveda, infertility

## Abstract

Infertility is encountered as a stressful condition by couples worldwide, impacting not just their physical and mental well-being but also placing financial strain on them. Ayurvedic management provides a promising, cost-effective avenue for addressing infertility disorders and enhances the success rates of in vitro fertilization (IVF), especially after previous unsuccessful attempts. This study aims to enhance clinical evidence and expand the scope of Ayurvedic approaches for managing infertility. A systematic literature search was conducted in PubMed and Scopus search engines for studies evaluating Ayurveda treatment modalities in infertility. Articles were searched using a combination of Medical Subject Heading (MeSH) terms, and the risk of bias was assessed using Robvis and the Joanna Briggs Institute (JBI) Critical Appraisal Tool. The review followed Preferred Reporting Items for Systematic Reviews and Meta-Analysis (PRISMA) guidelines. A total of 14 studies were considered in this systematic review, involving 248 patients. Among them, 84 were males and 164 were females. Of the 14 included studies, six were original studies, whereas eight were case reports. Our research contributes to addressing a notable research gap by conducting a comprehensive analysis of Ayurvedic treatments for infertility or medical conditions that lead to infertility. However, the limited sample size and lack of standardized protocols highlight the need for rigorous experimental research to establish the efficacy and safety of Ayurvedic treatments for infertility.

## Introduction and background

Infertility is a medical condition affecting either the male or female reproductive system, characterized by the inability to conceive despite having regular, unprotected sexual intercourse for 12 months or more [1]. The issue of infertility has received considerable critical attention worldwide. According to a recent World Health Organization (WHO) report, approximately 17.5% of the adult population, equivalent to one in six individuals globally, experience the impact of infertility. This emphasizes the crucial importance of making accessible, affordable, and high-quality fertility care available to those in need [2].

The inability to conceive is a distressing situation for couples all over the world. Infertility has a wide range of effects, including societal backlash and personal misery [3]. Addressing the issue of infertility requires crucial attention, as its impact extends beyond the physical inability to conceive, encompassing significant mental, emotional, and financial distress for individuals [4]. Although both men and women are responsible for infertility, societal perception often places a heavier burden of blame on women due to the traditional notion that women symbolize fertility [5].

Infertility causes are multifactorial in either the male or female reproductive system. Male infertility is commonly caused by issues with sperm, including reduced or absent sperm count, as well as abnormal sperm morphology and impaired sperm motility. Female infertility can result from a diverse array of abnormalities affecting the ovaries, uterus, fallopian tubes, endocrine system, and other factors [6]. Assisted reproduction technologies (ART) like in vitro fertilization (IVF), intrauterine insemination (IUI), and intra-cytoplasmic sperm injection (ICSI) offer effective infertility treatments, but they are often accompanied by high costs that pose a significant financial burden [7].

In Ayurveda, according to Susrutha, the essential factors for conception are mentioned as Ritu (reproductive period), Kshethram (female reproductive tract), Ambu (nutritional factors), and Beejam (sperm and ovum) [8]. Any abnormality or malformation of any of these harms the fertility outcome. Ayurveda takes into account an individual's constitution and aims to enhance the body's systems involved in fertilization.

The ayurvedic treatment of shodhana (purification) and shamana (balancing) therapies assists in eliminating blockages in the channels, pacifying imbalanced doshas, and facilitating the optimal formation of healthy semen (shukra dhatu) and ovum (stree shukra). This, in turn, promotes the chances of conception by creating a conducive environment for fertilization.

Shodhana refers to the purification or cleansing therapies in Ayurveda. It aims to eliminate the root cause of the disease by removing toxins or imbalances from the body. Mostly virechan (therapeutic purgation), vamana (therapeutic vomiting), and basti (therapeutic enema) are used in the treatment of infertility. Shamana refers to the palliative or pacifying therapies in Ayurveda. These treatments aim to alleviate symptoms, balance the doshas, and restore harmony to the body [9].

Ayurveda management of infertility has shown quite effective results in cases of infertility due to polycystic ovarian disorder (PCOD), tubal blockage, oligoasthenozoospermia, and others. However, there are limited reviews available that offer a comprehensive perspective on Ayurveda treatments for treating infertility. Thus, this systematic review aims to consolidate and analyze the published literature that explores the application of Ayurveda principles in treating infertility.

The primary objective is to lay a foundation for further research, strengthen clinical evidence, and broaden the horizons of Ayurvedic treatment in addressing infertility. Moreover, this study aimed to provide meaningful and applicable insights to healthcare practitioners who are considering the integration of Ayurvedic approaches in their clinical practice for infertility treatment.

## Review

Materials and methods

A systematic review of the literature was conducted to evaluate the efficacy of Ayurveda interventions in treating infertility. This review adheres to the Preferred Reporting Items for Systematic Reviews and Meta-Analysis (PRISMA) guidelines for the systematic review process.

PICO framework (population, intervention, control, and outcomes) was employed to outline the search strategy:

 a) Population: "infertile population"

 b) Intervention/exposure: "ayurveda treatment"

 c) Control: "Was not applicable in this study"

 d) Outcome: "efficacy assessment"

This review aimed to answer the following research question: " What is the effectiveness of Ayurvedic interventions in improving fertility outcomes?"

Literature Search and Identification of Studies

The Preferred Reporting Items for Systematic Reviews and Meta-Analysis (PRISMA) 2020 guidelines were used for this systematic review. A detailed literature search was performed on the PubMed and Scopus databases from January 2013 to December 2023 for studies evaluating Ayurveda treatment in infertility patients using the following Medical Subject Headings (Mesh) terms, "Medicine, Ayurvedic/adverse effects"[Mesh] OR "Medicine, Ayurvedic/instrumentation"[Mesh] OR "Medicine, Ayurvedic/methods"[Mesh] OR "Medicine, Ayurvedic/standards"[Mesh] OR "Medicine, Ayurvedic/statistics and numerical data"[Mesh] OR "Medicine, Ayurvedic/trends"[Mesh] AND "Infertility"[Mesh]. The following inclusion and exclusion criteria were considered (Table 1).

**Table 1:** Inclusion and Exclusion Criteria.

Inclusion criteria	Exclusion criteria
Studies conducted on human subjects.	Studies that are not conducted on humans, such as in vitro studies or animal studies.
Clinical studies, which may include randomized controlled trials, non-randomized trials, prospective and retrospective studies, cross-sectional studies, case series, and case reports dealing with Ayurveda treatment in infertile patients without any age or sex restriction, were included.	Research articles that did not involve the collection of original primary data, such as review articles, protocols, editorials, letters, and other non-original research publications.
Articles published in the English language between January 2013 and December 2023.	Articles published in languages other than English and before January 2013.
Accessible in full-text format.	Studies that fail to provide information on the outcomes of interest.

Study Selection

The titles and abstracts of the identified studies were assessed by two authors, and any disparity was resolved by a third author. Studies not assessing the efficacy of Ayurveda interventions on infertility were excluded. However, when the study abstract lacked clarity, the complete texts were obtained for assessment and independently analyzed by two authors.

Outcome Parameters

The primary outcome parameter was achieving conception following Ayurvedic treatment, while the secondary outcome parameter considered was the successful birth of a viable child.

Data Extraction

The data extracted included information on publication metrics, including the first author's name, publication year, and country, study design, sample size, characteristics of patients (sex, age), treatment plan, and study outcomes. The included studies were reviewed by two other authors. Due to the heterogeneity of the case reports and original studies, it was not possible to conduct a statistical evaluation of the results, and therefore, a meta-analysis was not performed.

Risk of Bias Assessment

The risk of bias assessment for case reports was appraised using the Joanna Briggs Institute (JBI) Critical Appraisal Tool for case reports [10]. This tool provides a JBI checklist for case reports. Eight questions were answered for each case report. The answers to questions were "yes," "no," "unclear," or “not applicable,” and each question scored 1 point for yes, 0 points for no, unclear, or not applicable; the corresponding score ranged from 0 to 8 [11]. A low risk of bias was assumed when the score was at least 7, and a high risk of bias was assumed when the score was less than 5. A moderate risk of bias was considered when the score was between 5 and 6.

The risk of bias for original studies was conducted using the R package and a Shiny web application, developed by the National Institute for Health Research (NIHR). This tool was part of the project under the Doctoral Research Fellowship (DRF-2018-11-ST2-048) at the University of Bristol, UK. The version from 2020 was employed for the analysis [12]. The program conducts assessments across six primary domains: 1) randomization methods, 2) the suggested intervention, 3) missing outcome data, 4) outcome measurement, 5) choice of reported outcomes, and 6) overall assessment.

Results

**Figure 1:**
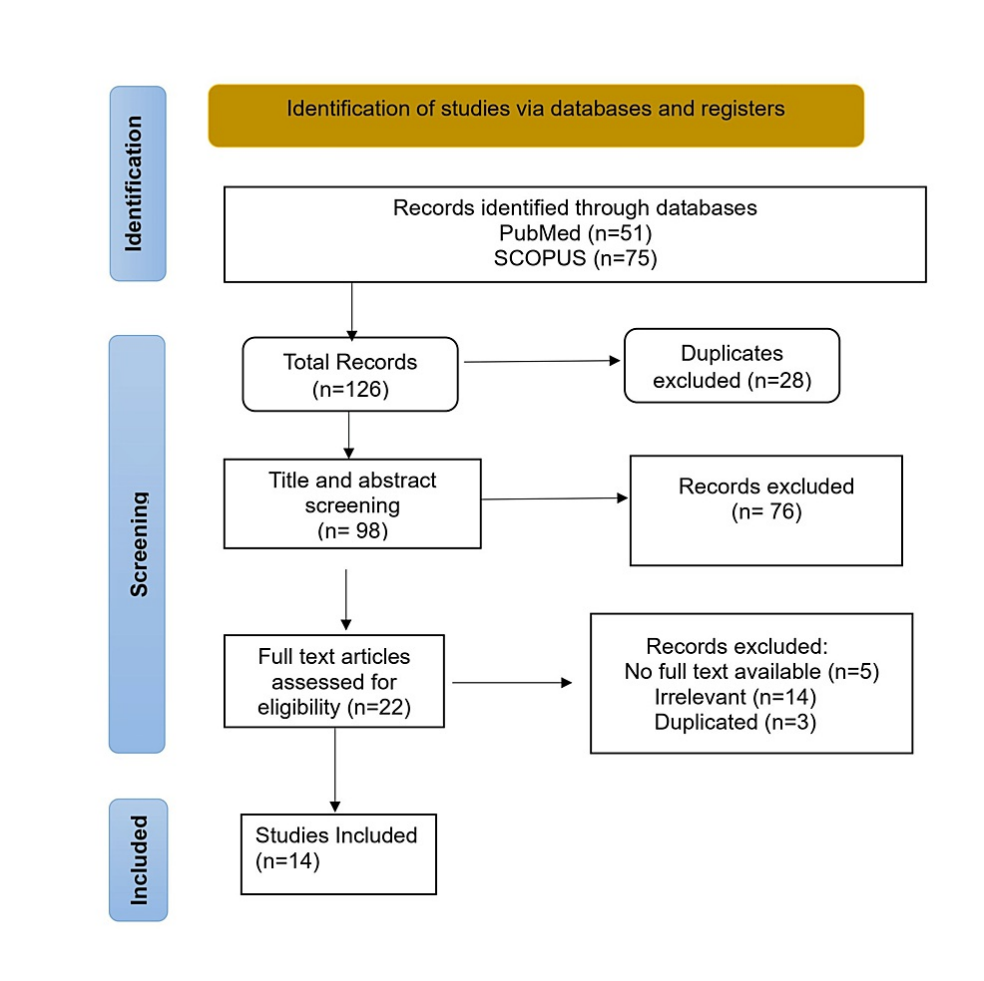
PRISMA Flowchart. PRISMA: Preferred Reporting Items for Systematic Reviews and Meta-Analyses.

A total of 14 articles meeting the inclusion criteria were selected in this review. The selection process adheres to the guidelines outlined in the Preferred Reporting Items for Systematic Reviews and Meta-Analyses (PRISMA). Figure 1 depicts the selection process.

Study Characteristics 

The selected articles included original studies and case reports published from 2013 to 2023. A detailed representation of the included studies is depicted in Table 2 [13-26].

**Table 2:** Detailed Representation of the Included Articles.

Author (s)/year	Country	Study design	Sample size	Age/Sex	Cause	Treatment Plan	Outcome
Ambiye et al., 2013 [13]	India	Randomized controlled trial (RCT)	46	Males, 22- to 40-year-old	Oligospermia	Group A (n=21): Ashwagandha root extract (KSM-66) 225 mg x 12 weeks. Group B (n=25): Placebo	Improvements in sperm parameters and serum testosterone in the ashwagandha-treated group after the 90-day treatment period
Hussain et al., 2018 [14]	Pakistan	Randomized controlled trial (RCT)	36	Males, 22 to 40-year-old	Oligospermia	Group A (n=23): Polyherbal formulation (PHF) 750 mg x 90 days. Group B (n=13): Placebo	Significant increase in sperm concentration, volume, motility, and serum levels of testosterone and luteinizing hormone
Baria et al., 2015 [15]	India	Open-label	19	Females, 19- to 38-year-old	Fallopian tube blockage	Yavakshara taila uttarabasti (5 ml) for 6 days with an interval of 3 days in between, after completion of menstrual cycle for two consecutive cycles	Tubal patency was achieved in 68.75% of patients, and conception was achieved in 6.25% of patients
Kumari et al., 2013 [16]	India	Open-label	100	Females, 20- to 35-year-old	Polycystic ovarian disease, tubal block, anovulation, and menstrual abnormalities	Prior sneha and shodhan basti, followed by uttarabasti with Dhanvantari taila for 5 days after completion of menstrual cycle	Overall conception rate was found to be 75%, with significant improvements observed in polycystic ovarian disease and tubal block cases
Donga et al., 2013 [17]	India	Parallel-arm	24	Females, 20- to 45-year-old	Anovulation	Group A (n=12): Treatment through Nasya (nasal administration) of Narayana taila (8 drops) x 14 days. Group B (n=12): Treatment with matra basti (rectal administration) of Narayana taila (60 ml) x 8 days	Group A: No patients conceived, and ovulation was observed in 36.36%. Group B: One patient conceived, higher ovulation rate of 66.16%
Rupareliya et al., 2021 [18]	India	Open-label	15	Females, 20- to 37-year-old	Thin endometrium	Saubhagyanandana ghrita yonipichu (vaginal tampon) 10 ml for 6 days after cessation of menses for one cycle and jeevaniya churna 10 g orally with milk twice before meals for 30 days	Improvement in endometrial thickness, menstrual cycle regularity, ovulation, and one patient conceived after the treatment
Vasudevan et al., 2021 [19]	India	Case Report	1	Male, 34-year-old	Oligoasthenozoospermia associated with anxiety	Counseling with lifestyle modification, vaishavanara choorna, snehapana with sukumara ghrita, snehana, and swedana. Shodhana chikitsa included virechan using gandharvahastadi eranda taila, and shamana chikitsa, with mashadi choornam shodhana chikitsa included virechan using gandharvahastadi eranda taila	Anxiety decreased from moderate to mild, increased sperm motility from 5% to 56%, decreased immotile sperm from 89% to 31%, and increased sperm concentration from 3 to 32 million/ml
Asmabi and Jithesh, 2022 [20]	India	Case Report	1	Female, 32-year-old	Polycystic ovarian syndrome (PCOS)	Herbal formulations along with shodhana chikitsa included vamana using madanaphala kalka and yashtimadhu phanta. Virechana using gandharvahastadi eraṇḍa taila. Basti-anuvasana basti using pippalyadi anuvasana taila and lekhana basti using erandamoola kwatha. Uttarabasti- using mahanarayana taila and shamana chikitsa with phalasarpis	A urine pregnancy test was positive within 8 months of treatment, and delivery of a healthy baby girl
Kesslera et al. 2015 [21]	Germany	Case Report	1	Female, 38-year-old	Idiopathic	Dietary and lifestyle modifications, herbal formulations (*Withania somnifera*, *Bacopa monnieri*, *Asparagus racemosus*, and *Tinospora cordifolia*), nutritional supplements, body massage, detoxification therapies	Conceived and delivered healthy baby boy.
Muraleedharan et al. 2018 [22]	India	Case Report	1	Female, 36-year-old	Low anti‑Mullerian hormone (AMH)	Vaisvanara churna, snehapana with sahanarayana taila, snehana and svedana (oleation and sudation), mriduvirecana with trivritlehyayoga basti (niruha basti with mustadi yapana basti and anuvasana basti with mahanarayana taila), and uttarabasti with mahanarayana taila	Improvement in AMH levels from 0.07 ng/ml to 2.11 ng/ml within the first 3 months of treatment
Doddamani et al., 2019 [23]	India	Case Report	1	Male, 56-year-old	Necrospermia	Shodhana chikitsa included virechana using eranda taila and shamana chikitsa with phala ghrita, chandraprabha vati, shilapravang vati, and arogyavardhini vati	After a 3½ month of treatment, increased sperm count from 2 to 9 million, increased sperm motility actively progressive from 0% to 30%, and nonmotile from 100% to 45%
Mehra et al., 2023 [24]	India	Case Report	1	Female, 31-year-old	Endometrial calcification	Deepana pachana with chitrakadi vati, mridu virechana with haritaki churna, and uttarabasti using kshara taila and phalaghrita, administered over a period of three months	Significant reduction in endometrial calcifications and regular menstrual cycles
Jadhav, 2022 [25]	India	Case Report	1	Female, 23-year-old	Large endometrioma	Warm, medicated massage (snehana), sudation (swedana), and both anuvasana and uttarabasti, along with yoga basti (eight medicated enemas) and Kuberaksha vati, followed by two sessions of yoga basti	The endometrioma size reduced, the patient conceived within four months and had a full-term normal delivery
Otta et al., 2021 [26]	India	Case Report	1	Female, 30-year-old	Tubal blockage	Snehapana followed by virechana (purgation) and Ayurvedic medications, phalaghrita, ashokarishta, kanchanara guggulu	Conceived after 16 weeks of treatment

Among the 14 studies included, which covered a total of 248 patients, two were randomized controlled clinical trials [13,14], three were open-label studies [15,16,18], one was a parallel-arm study [17], and eight [19-26] were case reports. The selected studies were carried out in India, Pakistan, and Germany (n=14). Twelve studies were conducted in India, and one each was conducted in Germany and Pakistan.

Two randomized controlled trials (RCTs) were conducted on males with oligospermia. One study [13] administered the root extract of ashwagandha in a capsule form, KSM-66, while the other RCT [14] provided a polyherbal formulation (PHF) that consisted of the root of *Chlorophytum borivilianum*, seeds of *Hygrophila spinosa *T. Anders, seeds of *Mucuna pruriens*, seeds of *Mimosa pudica*, sap of *Acacia senegal*, root of *Astragalus membranaceus*, seed coat of *Plantago ovata*, sap of *Bombax ceiba*, and root of *Eurycoma longifolia* and rocky candy. Two open-label studies involved administering Uttarabasti (medicated oil into the intrauterine cavity). One study [15] used Yavakshara taila, and the other study [16] utilized Dhanvantari taila. Moreover, a study [18] treated a patient with a thin endometrium using saubhagyanandana ghrita yonipichu (a vaginal tampon), while another study [17] administered Narayana taila to patients through two different routes of administration: nasal and rectal. The case reports [19-26] describe patients who were given treatment plans involving shodhana and shamana therapies, along with Ayurvedic medications, tailored to each patient.

Patient Demographics

The 14 studies that were selected included a total of 248 patients, with 164 being female and 84 males. The age range included males from 22 to 56 years and females from 19 to 45 years, covering a broad spectrum of infertility causes. For males, these causes included oligospermia and necrospermia, whereas for females, the issues were diverse, including fallopian tube blockages, polycystic ovarian syndrome, tubal obstructions, and anovulation.

Study Outcomes

The studies examined a variety of Ayurvedic treatments, including shodhana and shaman therapies, the use of ashwagandha root extract, polyherbal formulations, uttarabasti with medicated oils, and yonipichu (vaginal tampons soaked in medicated oil). The study outcomes were evaluated based on the improvement in the underlying medical condition causing infertility or successful conception. Two RCT studies focusing on oligospermia [13,14] showed significant improvement in sperm parameters and serum testosterone levels. The outcomes for female infertility were equally promising across a varied set of conditions, including fallopian tube blockage, polycystic ovarian disorder (PCOD), thin endometrium, and anovulation [15-18]. The study [15] showed successful conception and tubal patency in women with blocked fallopian tubes. Kumari et al. [16] study achieved a 75% conception rate, and another study [17], where the drug was administered through both nasal and rectal routes, ovulation occurred in both cases. However, the administration through the rectal route resulted in higher ovulation rates and conception. The case reports [19-26] of individual patients offer valuable insights into the potential benefits of Ayurvedic treatments across different causes of infertility. These reports not only demonstrate significant improvements in the medical conditions contributing to infertility but also highlight the successful conception achieved by most patients following Ayurvedic treatment.

Assessment of Risk of Bias

The risk of bias in selected case reports was independently evaluated by two authors using the Joanna Briggs Institute (JBI) critical appraisal checklist for case reports, and to address any potential disagreements, consultation with a third reviewer was undertaken for resolution. Out of eight included studies, seven scored 7 [19,20,22-26], whereas one obtained a score of 6 [21]. The risk of bias assessment is described in Table 3 [19-26].

**Table 3:** Risk of Bias Assessment Following the Joanna Briggs Institute (JBI) Critical Appraisal Tool for Case Reports.

Reference	Q1*	Q2*	Q3*	Q4*	Q5*	Q6*	Q7*	Q8*	Total Score
Vasudevan et al., 2021 [19]	Yes	Yes	Yes	Yes	Yes	Yes	No	Yes	7
Asmabi and Jithesh, 2022 [20]	Yes	Yes	Yes	Yes	Yes	Yes	No	Yes	7
Kesslera et al., 2015 [21]	Yes	Yes	Yes	No	Yes	Yes	No	Yes	6
Muraleedharan et al., 2018 [22]	Yes	Yes	Yes	Yes	Yes	Yes	No	Yes	7
Doddamani et al., 2019 [23]	Yes	Yes	Yes	Yes	Yes	Yes	No	Yes	7
Mehra et al., 2023 [24]	Yes	Yes	Yes	Yes	Yes	Yes	No	Yes	7
Jadhav,. 2022 [25]	Yes	Yes	Yes	Yes	Yes	Yes	No	Yes	7
Otta et al., 2021 [26]	Yes	Yes	Yes	Yes	Yes	Yes	No	Yes	7
*Details pertaining to Q1-Q8 are tabulated in Appendix 1.									

**Figure 2:**
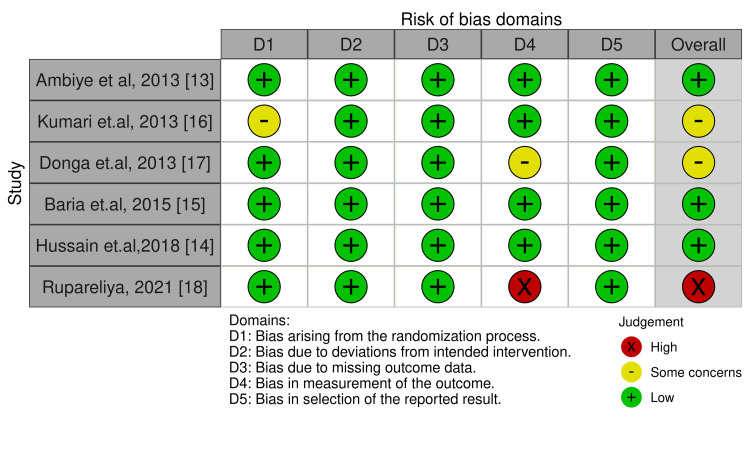
Illustration of the Risk-of-Bias Domains.

**Figure 3:**
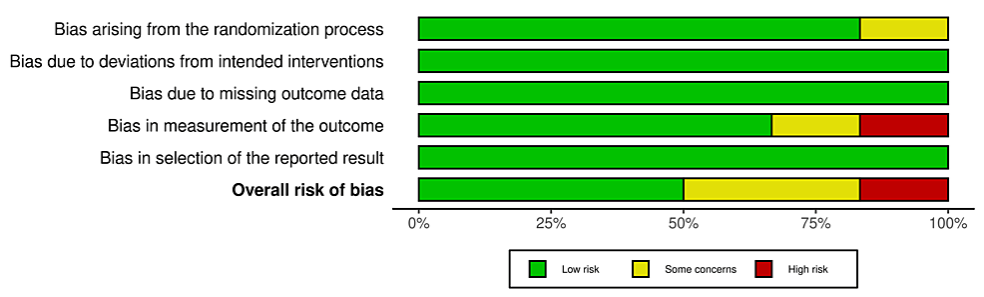
The Overall Risk of Bias From the Included Studies.

The risk-of-publication bias for original studies was achieved by using the R-based Robvis software package. Most of the domains showed a low risk of bias. Out of the six included studies, five studies (83.33%) showed a low risk or some concerns of bias [13-17]. Only one study (16.67%) showed high concerns [18], primarily due to bias in the measurement of the outcome. The risk of publication bias is represented in Figures 2, 3 [13-18].

The overall risk of bias from the included studies is depicted in Figure 3.

Discussion

Infertility is an escalating global health issue, with increasing rates observed worldwide. The impact of infertility extends beyond the physical realm, affecting the emotional and psychological well-being of couples. Furthermore, conventional treatment options, such as assisted reproductive techniques (ART), are often expensive and have variable success rates, imposing significant financial burdens on couples. Ayurveda, an ancient Indian system of medicine, offers a holistic approach to addressing infertility by focusing on improving overall health and balancing the body's doshas, thereby promoting overall reproductive health [27].

Ayurvedic interventions for treating infertility involve a combination of purification (shodhana) and pacification (shamana) therapies, along with lifestyle modifications and stress management tailored to individual patients. Therefore, Ayurveda holistically addresses infertility by enhancing overall health and improving the quality of life for individuals [28]. Prior research studies [29,30] have highlighted the beneficial effects of ashwagandha. Our findings corroborate these earlier observations, highlighting the effectiveness of ashwagandha in managing oligospermia [13].

Research on Ayurvedic preconception care improving ART outcomes in infertile couples has not been extensively researched or documented. A case study [22] showed the potential benefits of incorporating Ayurveda as a complementary treatment to enhance the success rates of IVF, particularly in instances where prior IVF attempts have been unsuccessful. For male infertility cases, studies [13,14,19,23] indicate that utilizing Ayurvedic therapies, including shodhana chikitsa and shamana chikitsa, or solely Ayurvedic herbal medicines, can enhance sperm count and motility [31]. These findings highlight Ayurveda's potential for managing male infertility and offer an alternative approach for those who are not willing to undergo assisted reproductive techniques.

In females, Ayurvedic management of infertility involved various Ayurvedic medications and shodhana therapies, especially basti and uttarabasti with medicated oils, which showed positive outcomes. The results showed improvements in menstrual cycles, hormonal balance, ovulation, successful natural conceptions, and the birth of healthy babies.

Overall, the findings from this review collectively support the efficacy of Ayurvedic interventions in managing various causes of infertility. The holistic nature of Ayurvedic treatments, which take into account an individual's constitution, lifestyle, and emotional well-being, may also play a role in reducing anxiety and stress levels associated with infertility.

However, it is important to note that a key limitation of this review is the heterogeneity of study designs and the variance in sample sizes, which may impact the generalizability of the findings. Additionally, it is essential to recognize that the findings, primarily derived from case studies involving a limited number of participants, might be susceptible to bias. This underscores the need for careful interpretation in future research. Furthermore, the lack of standardized treatment protocols in Ayurveda poses challenges in replicating and validating the results across different populations.

Future research should aim at conducting large-scale, randomized controlled trials to establish standardized, evidence-based Ayurvedic treatment protocols for infertility. Additionally, exploring the mechanisms of action of Ayurvedic treatments and their interactions with conventional fertility treatments could provide deeper insights into integrated approaches for infertility management.

## Conclusions

This systematic review emphasizes the promising potential of Ayurvedic treatments for infertility, highlighting positive outcomes in sperm quality, conception rates, and overall reproductive health through traditional Indian medicine practices. Incorporating Ayurveda into infertility treatment strategies offers a natural, safe, and cost-effective option for couples facing infertility. Furthermore, merging Ayurvedic principles with conventional fertility treatments presents a holistic approach, suggesting a valuable complement to assist reproductive technologies. However, future studies should focus on larger, more rigorous trials to confirm these findings and explore integrated fertility care.

## Appendices

**Table 4:** JBI Critical Appraisal Checklist for Case Reports JBI: Joanna Briggs Institute.

S No.	Questions	Description
1	Q1	Were the patient’s demographic characteristics clearly described?
2	Q2	Was the patient’s history clearly described and presented as a timeline?
3	Q3	Was the current clinical condition of the patient on presentation clearly described?
4	Q4	Were there diagnostic tests or assessment methods, and were the results clearly described?
5	Q5	Was the intervention(s) or treatment procedure(s) clearly described?
6	Q6	Was the post-intervention clinical condition clearly described?
7	Q7	Were adverse events (harms) or unanticipated events identified and described?
8	Q8	Does the case report provide takeaway lessons?
